# Intercultural communication competence and job burnout in MNC employees: the mediation role of job stress

**DOI:** 10.3389/fpsyg.2024.1339604

**Published:** 2024-03-19

**Authors:** Xiaoxia Xie, Yulu Tu, Chienchung Huang

**Affiliations:** ^1^Research Institute of Social Development, Southwestern University of Finance and Economics, Chengdu, China; ^2^Faculty of International Studies, Southwestern University of Finance and Economics, Chengdu, China; ^3^School of Social Work, Rutgers University, New Brunswick, NJ, United States

**Keywords:** Chinese multinational corporation, intercultural communication competence, job burnout, job stress, Southeast Asia

## Abstract

This study examined the relationship between intercultural communication competence (ICC) and job burnout, as well as the mediating effects of job stress, using data collected from employees (*n* = 1,064) from a Chinese multinational corporation in Brunei. Through regression analysis and mediation effect tests, we found that ICC was negatively associated with job burnout (β = −0.19, *p* < 0.001) and job stress (β = −0.08, *p* < 0.001). Job stress was positively associated with job burnout (β = 0.65, *p* < 0.001). Job stress played a partial mediating role between ICC and job burnout. The total effect of ICC on job burnout was −0.19, the direct effect was −0.14, and the indirect effect of ICC via job stress was −0.05. The findings call for ICC training for employees in multinational corporations.

## 1 Introduction

Over the past 50 years, Southeast Asian economies have transformed from primarily agrarian societies toward more modern industrial states (Benson and Yukongdi, 2005). The Southeast Asian region involves the following countries: Brunei, Cambodia, Indonesia, Laos, Malaysia, Myanmar, the Philippines, Singapore, Thailand, East Timor, and Vietnam (De Cieri and Bardoel, 2009). Inevitably, countries in this region are interrelated with China in various ways, especially in economic and cultural aspects. Nevertheless, the remarkable growth of the Chinese economy has generated a high rate of employee turnover in Asia, especially in Asian multinational firms (Zheng and Lamond, 2010). However, studies explaining the occupational status of employees in this region are quite limited compared to European- or American-based multinational corporations (MNCs).

Company H is located in Brunei and headquartered in China. In 2022, the company experienced a high turnover rate, reaching 20.8%, while the turnover rates of other subsidiaries were less than 4% within Chinese borders. If employees show high resignation intentions with high frequency, the company will suffer from monetary, reputational, and opportunity losses (Black and Mendenhall, 1989; Graf, 2004; Okpara et al., 2021). This shows researchers’ interest in employees’ stability, its antecedents, and its outcome.

According to the Ministry of Commerce of the People’s Republic of China, the annual non-financial direct overseas investment of Chinese enterprises in the countries along the Belt and Road (B&R) initiative reached 141.05 billion yuan (20.97 billion US dollars) in 2022.^1^ The Southeast Asian region is the main target investment region, including Singapore, Indonesia, Malaysia, Thailand, Vietnam, Cambodia, and Brunei (see Footnote 1). By the end of 2021, more than 11,000 enterprises had been established in B&R countries, accounting for a quarter of China’s total overseas enterprises.^2^ Total registered overseas subsidiary corporations with parent companies set up in China reached 48,828 on April 13, 2023.^3^ In the meantime, an increasing number of laborers have been expatriated. For example, in 2022, Chinese enterprises assigned 259,000 laborers abroad, and 543,000 laborers stayed abroad at the end of the year.^4^ In addition, according to a thorough statistical analysis of the average overseas assets and income of China’s top 100 multinational companies from 2011 to 2022, Zhang (2023) found that their average assets increased from 32.5 billion Yuan (approximately 4.75 billion US dollars) in 2011 to 107.5 billion Yuan (approximately 15.72 billion US dollars) in 2022, a sharp rise of 230.8%; the average revenue of the top 100 MNCs increased from 31 billion Yuan (approximately 4.53 billion US dollars) in 2011 to 77.9 billion Yuan (approximately 11.39 billion US dollars) in 2022, a notable increase of 151.3%. It means the past 10 years have witnessed a booming development of Chinese multinational companies.

Company H is one of the top 100 Chinese MNCs. Despite its large investment scale (3.45 billion US dollars) and assets (6.83 billion US dollars), its turnover rate reached 20.1% in 2022, which is quintuple as much as domestic companies (3.9%). Thus, it is urgent to investigate the employees’ occupational status.

Job burnout turns out to be strongly and positively correlated with turnover intentions (Houkes et al., 2001; Mor Barak et al., 2001; Kim and Stoner, 2008; Choi et al., 2012; Ran et al., 2020). While a large body of empirical research lays emphasis on job burnout within one single country, such as social workers (Huang et al., 2021, 2022), healthcare staff (Ran et al., 2020), teachers (Azeem and Nazir, 2008), and other working areas (e.g., telecom, IT, business, corporate, and sports) (Maslach and Jackson, 1984; Maslach and Schaufeli, 1993; Maslach, 2006), burnout research has expanded beyond its original borders and is expanding internationally (Maslach et al., 2001; Sweileh, 2020). However, there are limited studies aiming at employees’ job burnout among joint ventures and MNCs overseas and under intercultural backgrounds, especially in Chinese-based firms. With the rapid growth of foreign investment from Chinese enterprises, it is prudent to investigate the staff’s job burnout status with an eye on their cross-cultural competence.

Hence, to fill the literature gap, this study selected a Chinese MNC (Company H) located in Brunei, a Southeast Asian country, along the Belt and Road initiative, using a questionnaire to empirically investigate employees’ job burnout status, the relationship between intercultural communication competence and job burnout, as well as the mediating role of job stress.

## 2 Literature review, theory, and conceptual framework

### 2.1 Job burnout

Job burnout is a psychological syndrome that indicates a prolonged reaction to workplace stressors, and it mainly refers to the chronic strain that is attributed to an incongruence, or misfit, between the employee and the job (Maslach, 2003, p. 189). It includes three key dimensions: exhaustion, cynicism, and a sense of inefficacy. Since Maslach and Jackson (1981) developed the concept and measurement tools for job burnout, the trajectory of burnout research has dealt with people’s real experiences in the workplace, which led to a comprehensive understanding of the context and outcomes of this phenomenon as well as intervention strategies. For example, burnout researchers have focused more on job context, such as job demands and resources (Bakker et al., 2014; Huang et al., 2021; Xie et al., 2021), and social support (Kahn et al., 2006; Xie et al., 2022), than on individual variables such as personality (Maslach, 2003). As for its outcomes, burnout is strongly and positively associated with turnover and plays a negative role in employees’ occupational performance (Randall and Scott, 1988; Rahim and Cosby, 2016; Wu et al., 2019). For MNCs, employees’ engagement is essential for the smooth running of the overseas business, either expatriates or local staff (Kumar and Pansari, 2014; Rodrigues da Costa et al., 2019).

### 2.2 Intercultural communication competence, job stress, and burnout

Despite the inescapability of cross-cultural communication in the daily work of MNC workers, little is known about how their intercultural communication competence affects burnout. Intercultural communication is often defined as “communication between people from different national cultures, and many scholars limit it to face-to-face communication” (Gudykunst, 2002, p. 179). Since Hall (1959) laid the foundation of the intercultural communication research area, much progress has been made, and one of the prominent subjects of interest to researchers is intercultural communication competence (Arasaratnam and Doerfel, 2005). ICC remains an important focus of intercultural scholars and practitioners (Martin, 2015) and is involved in the practical application of intercultural communication in situations such as pluricultural classroom (Holmes, 2006), training (Kupka et al., 2008), sojourning (Jackson, 2009), expatriates (Peltokorpi, 2010; Wilczewski et al., 2018), and participating in daily intercultural interactions in an increasingly multicultural society (Taylor and Osland, 2012; Balakrishnan et al., 2021). In multicultural groups, many, if not all, obstacles can be traced to the diverse cultural contexts of group members, such as cultural diversity and relational, communication, and cultural orientation differences (Matveev and Milter, 2004; Izogo and Mpinganjira, 2020). Abundant studies have proved that intercultural communication competence affects performance within multicultural teams (Matveev and Nelson, 2004; Abugre and Debrah, 2019) and expatriates’ adaptation and adjustment (Brein and David, 1971; Abugre, 2018), which is vital for MNC sustainable operation. Overall, competency in intercultural interactions has become a necessity for individuals to succeed in a multicultural environment.

As for the studies on intercultural communication competence and burnout, scholars seldom tested their relationship among MNC employees or expatriates, as most of them chose health and human service areas, such as nurses and healthcare providers. Ulrey and Amason (2001) found a negative association between healthcare providers’ intercultural communication effectiveness and their levels of anxiety in a survey of the employees of a large healthcare system, which included two hospitals and four clinics. Lee et al. (2022) conducted a survey on 146 nurses working in the operating room (OR) in South Korea and verified that communication competence was adversely associated with burnout among OR nurses after controlling for other factors related to burnout. Babin et al. (2012) examined burnout from a communication perspective and found communication competence will predict the extent to which participants report experiencing job burnout. For burnout researchers, the emphasis is on the context of the job environment, such as workload demands and social support from colleagues (Maslach, 2003). Intercultural communication can be deemed as one of the demands and resources of multinational company jobs, which requires attention in the burnout research field.

In contrast to the paucity of literature connecting ICC to job burnout, a large body of literature exists that explores the relationship between job stress and burnout across a variety of work-related contexts (e.g., Innstrand et al., 2004; Leung et al., 2011; Silbiger and Pines, 2014; Mullen et al., 2018; Freitas et al., 2023). Stress can be defined as “a nonspecific response of the body to any demand” (Seyle, 1983, p. 127). The concept of stress has also been extended to the context of work. Parker and DeCotiis (1983, p. 165) defined job stress as “feeling of a person who is required to deviate from normal or self-desired functioning in the workplace as the result of opportunities, constraints, or demands relating to potentially important work-related outcomes.” Stressful working environments may generate physical, psychological, and professional consequences for individuals. For physical consequences, one may suffer from physical injuries, cardiovascular disease, and high blood pressure (Mohammad Mosadeghrad, 2014), or even severe, premature death and disability along with chronic suffering from job stress (Quick and Henderson, 2016). For psychological impact, constant stress may lead to depression (Freudenberger, 1989; Liu et al., 2012; Shen et al., 2014) and anxiety (Nyssen et al., 2003; Mark and Smith, 2012). For professional aspect, job satisfaction (Dartey-Baah et al., 2020), organizational commitment (Wong et al., 2021), and job burnout are common symptoms due to job stress. Abundant research has found that job stress is the antecedent of burnout (Nyssen et al., 2003; Leung et al., 2010; Li et al., 2015; Luo et al., 2016; Mnif and Rebai, 2022; Freitas et al., 2023).

Intercultural communication can be a stressor for employees in pluricultural working settings. Job stressors are varied in different aspects, from relational labor and emotional labor to work duties such as interpersonal conflicts and a lack of training or competency. In particular, the misfit or fit between an employee’s job demands and his or her ability to meet such demands is a critical issue in determining whether stressors will trigger a stress response (Mullen et al., 2018). Intercultural research has shown that communicating with people from different cultures is one trigger that often brings anxiety and stress (Olaniran, 1993; Redmond and Bunyi, 1993; Gilstrap et al., 2019). Similarly, some scholars find that intercultural communication contributes to stress for healthcare providers (Kreps and Kunimoto, 1994; Schott and Henley, 1996). This is also the case for employees working in MNCs or being expatriated (Silbiger and Pines, 2014; Wilczewski et al., 2018).

### 2.3 Conceptual framework

**Figure 1:**
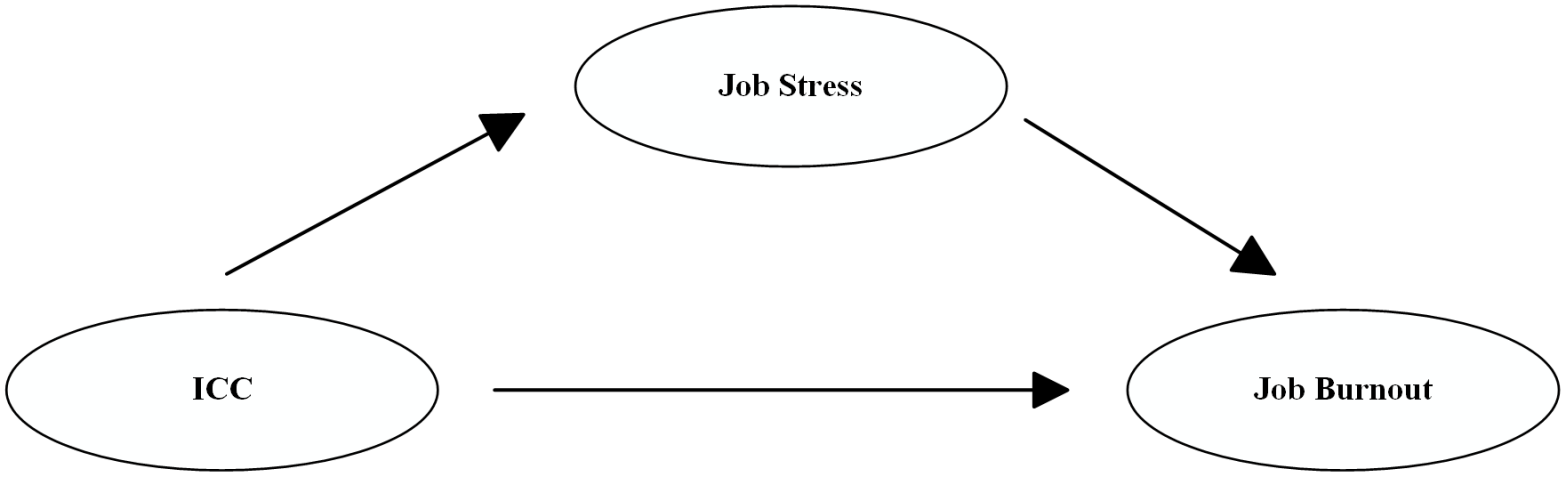
Conceptual model of intercultural communication competence, job stress, and job burnout.

Overall, empirical research on the relationship among intercultural communication competence, job stress, and job burnout among Chinese MNC workers is still lacking. Thus, this study constructs a model of the relationship between Chinese MNC employees’ intercultural communication competence, job stress, and job burnout (see Figure 1), proposing the following hypotheses:

*H1:* Intercultural communication competence is negatively associated with job burnout.

*H2:* Job stress is positively related to job burnout.

*H3:* Job stress plays a mediating role between intercultural communication competence and job burnout.

## 3 Methods

### 3.1 Data and sample

The data for the present study were collected from an anonymous survey administered to employees from Company H in Brunei Darussalam, a Southeast Asian country along the Belt and Road Initiative. Company H is an energy-based enterprise and a subsidiary of a large private enterprise in China. In 2017, the company built a refining and chemical joint venture with the Brunei government to make the best use of Brunei’s rich oil and gas resources and superior investment environment to implement international production capacity cooperation. Currently, there are approximately 1,000 Chinese employees and 500 local employees in Company H.

The official invitation to participate in the survey was sent to all employees in Company H on November 30, 2022. Two reminders to participants were spread the first week and second week via Company H’s official email and enterprise WeChat group. The online survey was conducted via the Wenjuanxing Platform in both Chinese and English versions, which was verified by all the authors and the English speakers in the Human Resource Department from Company H. On December 14, 2022, a total of 1,064 employees responded to the survey, and all the data were complete and valid, including Chinese, Bruneian, and Malaysian employees. The response rate of the survey was 70.9%. For ethical issues, all participants were informed that their participation was in an anonymous and voluntary way and that they could choose to stop the survey at any time.

### 3.2 Measures

#### 3.2.1 Job burnout

The Maslach Burnout Inventory (MBI) is the most authoritative and commonly used scale for job burnout (Maslach and Jackson, 1981; Maslach and Jackson, 1986). The MBI-GS (Maslach Burnout Inventory-General Survey) was designed in 1996 by Maslach, Jackson, and Leiter and was applied to occupational groups other than human services and education, such as customer service, maintenance, manufacturing, management, and most other professions. The MBI-GS addresses three dimensions: exhaustion, cynicism, and professional efficacy. Exhaustion measures feelings of being overextended and exhausted by one’s work. Cynicism measures an indifference or a distant attitude toward work. Professional efficacy measures satisfaction with past and present accomplishments, and it explicitly assesses an individual’s expectations of continued effectiveness at work if they are not effective at work.

In 2002, Professor Li Chaoping was authorized by the developer of the questionnaire, Professor Michael Leiter, to revise the MBI-GS in China. The results show that the inventory has good reliability and validity in China (Li and Shi, 2003; Zhong et al., 2009). Thus, this study adopted the Chinese version of MBI-GS for Chinese employees and used the original English version for Bruneian employees. The scale consists of 15 items, and responses to items in MBI-GS range from 0 (never) to 6 (every day). We reversed the item scores in the personal accomplishment subscale so that higher scores indicated greater burnout. The higher the total score, the more severe the job burnout. In this research, Cronbach’s alpha coefficient of the job burnout scale was 0.90.

#### 3.2.2 Job stress

The Simple Occupational Stress Inventory (SOSI) uses nine items to measure the level of occupational stress from two perspectives: time stress and anxiety stress. SOSI is an abbreviated version of the Parker and DeCotiis (1983) Occupational Stress Questionnaire, revised by Jamal and Baba (1992). There are 13 items in the original scale, and SOSI is simplified into nine items. The translation was published, and the Chinese version was adopted directly without modification in a published translation (Fields, 2004). Scholars have proved the validity of the SOSI Chinese version (Zhang, 2012). Zhang (2012) extracted two common factors in nine items, explaining 55.3% of the total variation, and the homogeneity reliability α coefficient and split-half reliability *r* coefficient were both >0.70. The scale’s measurement dimensions, time stress and anxiety stress, refer to an individual’s continuous pressure under the job (four items) and work-related anxiety (five items), respectively. In this research, Cronbach’s alpha coefficient of the job stress scale was 0.90. Responses to items in SOSI range from 1 (strongly disagree) to 5 (strongly disagree). Higher scores indicated greater stress.

#### 3.2.3 Intercultural communication competence

The Intercultural Communication Competence (ICC) scale was developed by Maslach et al. (1996); Arasaratnam (2009) to measure the intercultural communication competence of individuals and includes 10 items. ICC was a unidimensional construct and was used as the independent variable in this study. The Cronbach’s alpha reliability coefficients of this scale were 0.77 (Arasaratnam, 2016). Respondents were asked to rate their responses, such as “I often find it difficult to differentiate between similar cultures,” and “I usually look for opportunities to interact with people from other cultures.” Responses to items in SOSI range from 1 (strongly disagree) to 5 (strongly disagree). A higher score points to better intercultural communication competence. In this research, Cronbach’s alpha coefficient of the ICC scale was 0.70.

#### 3.2.4 Control variables

Age, sex, nationality, education, marital status, number of children, and salary were selected as control variables. The selected control variables are general characteristics and were found to be related to job burnout (Bhanugopan and Fish, 2006; Lee et al., 2022). For measuring age, participants were asked to fill “the year of your birth” question. For sex, we assigned 0 = female, 1 = male; for nationality, we assigned 0 = Chinese, 1 = Bruneian; for education, 0 = below college, 1 = college and above; for marital status, 0 = unmarried, 1 = married. As for age, number of children, and salary, they were numeric variables.

### 3.3 Analytical strategy

First, descriptive analysis and Pearson’s correlation analysis were adopted to overview the sample characteristics and correlations among all variables. Then, we conducted regression analysis and bootstrap analysis to examine the relations among ICC, job stress, and job burnout while controlling age, sex, religion, education, marital status, number of children, and salary. Finally, bootstrapping is used to verify the indirect effects and confidence intervals to further examine the explanatory power of the results. Bootstrap analysis can obtain a more accurate confidence interval and a higher test power (Hayes and Scharkow, 2013; Kristopher, 2014), compared with the stepwise method of Baron and Kenny (1986). STATA software 16.0 was used for all analyses.

## 4 Results

Table 1 presents the descriptive statistics and correlations of the key variables. In total, 676 Chinese employees, 379 Bruneian employees, and nine Malaysian employees participated in the survey. The nine Malaysian employees were combined into Bruneian employees as they share a similar cultural background and religious beliefs. The average burnout score of employees was 39 (SD = 22.7), but the average burnout score for Bruneian workers reached a high level of 52. The sample average score of job stress was 23.8 (SD = 7.8) and 31.6 (SD = 3.4) for ICC. About 86% of the sample was male. The average age of the sample was 35.9, and approximately 65% of them had at least a college degree. Approximately 60% of participants are married, and 35% have a particular religious belief. In addition, the sample average salary is RMB 14,425.7 per month (approximately 2,024 US dollars).

**Table 1:** Descriptive statistics and correlations of key variables.

Variable	Mean (SD)	1	2	3	4	5	6	7	8	9	10
1. ICC (intercultural communication competence)	31.63 (3.43)	1.000									
2. Job stress	23.75 (7.80)	−0.087***	1.000								
3. Job burnout	38.99 (22.69)	−0.208***	0.703***	1.000							
4. Age	35.86 (8.67)	0.097**	−0.341***	−0.395***	1.000						
5. Sex (0 = female,1 = male)	0.86 (0.34)	−0.030	−0.270***	−0.245***	0.205***	1.000					
6. Nationality (0 = Chinese, 1 = Bruneian)	0.36 (0.48)	−0.015	0.601***	0.444***	−0.418***	−0.328***	1.000				
7. Education (0 = below college, 1 = college and above)	0.65 (0.48)	0.034	0.060	0.117***	−0.281***	−0.129***	−0.020	1.000			
8. Marital status (0 = unmarried, 1 = married)	0.60 (0.49)	0.027	−0.253***	−0.307***	0.533***	0.185***	−0.316***	−0.230***	1.000		
9. Number of children	0.88 (0.91)	−0.004	−0.260***	−0.321***	0.493***	0.185***	−0.294***	−0.325***	0.707***	1.000	
10. Salary (RMB)	14,425.7 (7,640.9)	0.085**	−0.372***	−0.317***	0.485***	0.156***	−0.549***	0.222***	0.305***	0.202***	1.000

*N* = 1,064. *p < 0.05, **p < 0.01, ***p < 0.001.

The correlations of the key variables are presented in Table 1. Age was positively correlated with ICC (r = 0.10, *p* < 0.001) and negatively correlated with job stress (r = −0.34, *p* < 0.001) and job burnout (r = −0.40, *p* < 0.001). In addition, higher education was positively correlated with job burnout (r = 0.12, *p* < 0.001). In addition, employees with married status showed lower job stress (r = −0.25, *p* < 0.001) and lower job burnout (r = −0.31, *p* < 0.001). In addition, the number of children also had a negative effect on job stress (r = −0.26, *p* < 0.001) and job burnout (r = −0.32, *p* < 0.001). What’s more, salary showed a positive relationship with ICC (r = 0.09, *p* < 0.01) and a negative relationship with job stress (r = −0.37, *p* < 0.001) and job burnout (r = −0.32, *p* < 0.001). What’s more, employees with higher salary showed higher ICC, lower job stress, and lower job burnout status. In general, ICC was negatively correlated with job burnout (r = −0.21, *p* < 0.001) and job stress (r = −0.09, *p* < 0.001). Job stress is strongly and positively correlated with job burnout (r = 0.70, *p* < 0.001). That is, the higher the job stress of the employee was, the more likely the job burnout was to increase.

Table 2 presents the standardized estimates of the regression results. Four models were presented. Model 1 analyzed how ICC affected job burnout. The results showed that adjusted *R*^2^ = 0.30, *F* = 58.38, *p* < 0.001; ICC was negatively associated with job burnout (β = −0.19, *p* < 0.001), which meant if employees had higher intercultural communication competence, their job burnout level would be lower. Model 2 presented the effect of job stress on job burnout. The results show that adjusted *R*^2^ = 0.53, *F* = 148.99, *p* < 0.001; job stress played a significantly positive role on job burnout (β = 0.65, *p* < 0.001). Higher job stress leads to higher job burnout. Model 3 analyzed how ICC impacted job stress. The results showed that adjusted *R*^2^ = 0.38, *F* = 82.74, *p* < 0.001; ICC was negatively related to job stress (β = −0.08, *p* < 0.001). That is, if employees possessed higher ICC, their job stress would be lower. Model 4 presented the effect of ICC and job stress on job burnout (*R*^2^ = 0.55, *F* = 143.51, *p* < 0.001), pointing out that the fitness of Model 4 is higher than that of Model 1. ICC was negatively associated with job burnout (β = −0.14, *p* < 0.001), and job stress is positively associated with job burnout (β = 0.63, *p* < 0.001). In conclusion, Model 1 verified Hypothesis 1, indicating that intercultural communication competence has a negative association with job burnout. Hypothesis 2 was proved by Model 2, that is, the level of job stress has a positive effect on job burnout.

**Table 2:** Regression analysis of job burnout.

	Model 1	Model 2	Model 3	Model 4
	Job burnout	Job burnout	Job stress	Job burnout
	β (*SE*)	β (*SE*)	β (*SE*)	β (*SE*)
ICC (intercultural communication competence)	−0.191***		−0.075**	−0.143***
	(0.171)		(0.055)	(0.139)
Job stress		0.647***		0.631***
		(0.078)		(0.077)
Age	−0.137***	−0.127***	−0.044	−0.127***
	(0.095)	(0.075)	(0.031)	(0.077)
Sex (0 = female, 1 = male)	−0.089***	−0.038^+^	−071**	−0.044*
	(1.812)	(1.495)	(0.586)	(1.465)
Nationality (0 = Chinese, 1 = Bruneian)	0.293***	−0.048	0.518***	−0.036
	(1.549)	(1.430)	(0.501)	(1.404)
Education (0 = below college, 1 = college and above)	0.047	0.011	0.047^+^	0.013
	(1.445)	(1.190)	(0.468)	(1.166)
Marital status (0 = unmarried, 1 = married)	−0.011	−0.024	−0.019	−0.021
	(1.774)	(1.460)	(0.574)	(1.429)
Number of children	−0.120**	−0.074*	−0.061	−0.079**
	(0.946)	(0.779)	(0.306)	(0.763)
Salary/1,000	−0.042	−0.015	−0.053	−0.004
	(0.106)	(0.087)	(0.034)	(0.086)
_cons	95.208***	11.845*	29.420***	41.291***
	(6.379)	(3.706)	(2.017)	(5.468)
*N*	1,064	1,064	1,064	1,064
*F*	58.38***	148.99***	82.74***	143. 51***
Adjusted *R*^2^	0.302	0.527	0.381	0.547

^+^*p* < 0.10, *p < 0.05, **p < 0.01, ***p < 0.001.

Furthermore, among the control variables, age, sex, nationality, and number of children passed the significant test in most of the four models, suggesting that these four variables were associated with job stress and burnout. For instance, in Model 1, age (β = −0.14, *p* < 0.001), male (β = −0.09, *p* < 0.001), and number of children (β = −0.12, *p* < 0.001) were negatively associated with job burnout, while nationality (Bruneian) (β = 0.29, *p* < 0.001) was positively correlated with job burnout.

### 4.1 Partial mediating role of job stress

**Figure 2:**
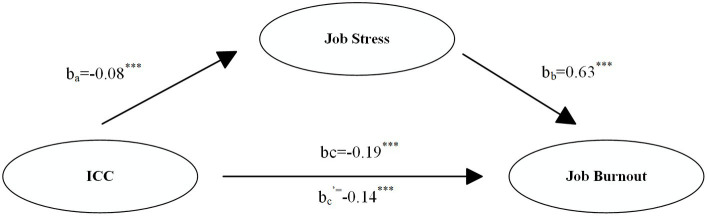
Conceptual model of intercultural communication competence, job stress, and job burnout.

By controlling age, sex, nationality, education, marital status, number of children, and salary, this article tested the mediation effect of job stress between ICC and job burnout. Table 3 and Figure 2 have suggested that after adding the intermediary variable job stress, ICC played a significant negative effect on job burnout (β = −0.21, *p* < 0.001), and the coefficient effect on job burnout was less than the original −0.19. The total effect of ICC on job burnout was −0.19, the direct effect was −0.14, and the indirect effect of ICC via job stress was −0.05 (*p* < 0.001), as presented in Table 3 and Figure 2. In this case, job stress had a partial mediating effect on job burnout. The mediating effect was −0.05, the 95% bootstrap confidence interval was [−1.241, −0.667], and the proportion of the effect mediated by job stress was −0.05 (b_a_ × b_b_). Thus, Hypothesis 3 was verified: the level of job stress plays a mediating role between intercultural communication competence and job burnout.

**Table 3:** Bootstrap analysis of mediation effect test.

Path	Observed coefficients	*SE*	95% Bootstrap confidence interval
ICC → job stress → job burnout	−0.95	0.147	[−1.241, −0.667]

## 5 Discussion

Currently, empirical research lays emphasis on job burnout within one single country (Azeem and Nazir, 2008; Ran et al., 2020; Huang et al., 2021); few studies aim at employees’ job burnout among joint ventures or MNCs overseas and under intercultural background. However, burnout research has expanded beyond its original borders and is expanding internationally (Maslach et al., 2001; Sweileh, 2020). Therefore, the present study extends the literature by investigating employees’ job burnout status in a large Chinese MNC located in Brunei, a Southeast Asian country.

In this study, intercultural communication competence was found to play a significant role in MNC employees’ job burnout in regression analysis. The results showed that a higher level of intercultural communication competence was associated with a lower level of job burnout, which echoes that, although scholars seldom directly tested the relationship between ICC and job burnout, previous research that verified communication competence could release the job burnout level. For example, Lee et al. (2022) conducted a survey on 146 nurses working in the operating room (OR) in South Korea and verified that communication competence was adversely associated with burnout among OR nurses after controlling for other factors related to burnout. Babin et al. (2012) found that communication competence will predict the extent to which participants report experiencing job burnout. In their daily work, cross-cultural communication among MNC workers is inevitable. Obstacles can be traced to the diverse cultural contexts of group members, such as cultural diversity and relational, communication, and cultural orientation differences (Matveev and Milter, 2004; Izogo and Mpinganjira, 2020). Abundant studies have proved that intercultural communication competence affects performance within multicultural teams (Matveev and Nelson, 2004; Abugre and Debrah, 2019) and expatriates’ adaptation and adjustment (Brein and David, 1971; Abugre, 2018), which is vital for MNC sustainable operation. Competency in intercultural interactions has become a necessity for individuals to succeed in a multicultural working environment, so the ICC level that an employee possesses can affect his/her job burnout level.

Importantly, job stress was proven to act as a mediator between ICC and job burnout. On the one hand, ICC was negatively related to job stress, which was consistent with the existing research results (Redmond, 2000; Ulrey and Amason, 2001). Ulrey and Amason (2001) found effective intercultural communication can lower healthcare providers’ anxiety so as to reduce their stress in a survey of the employees of a large healthcare system. Redmond (2000) also found a negative relationship between the handling of stress and intercultural communication competence, as reported by international students attending a US university. On the other hand, job stress played a significantly positive role in job burnout, which has been proven by a variety of empirical research in work-related contexts (e.g., Innstrand et al., 2004; Leung et al., 2011; Silbiger and Pines, 2014; Mullen et al., 2018). In this study, higher ICC can reduce employees’ job stress and effectively lower the job burnout that occurs at work.

Based on the findings presented above, it is suggested to improve employees’ intercultural communication competence in MNCs. In company H, Bruneian staff can speak English and Malay, while only 10% of Chinese staff can speak fluent English. To improve intercultural communication competence, Chinese staff are suggested to strengthen language competence first, such as spoken English, so as to eliminate language barriers. Second, intercultural activities, such as team building, ice-breaking games, and celebrating traditional festivals both from China (such as the Spring Festival) and Brunei (such as Raya), are encouraged. Third, the findings call for ICC training for employees to broaden their cross-cultural view of multinational corporations.

The results of this study must be considered with several limitations. First, the application of a cross-sectional dataset only allows us to approximate associative relations, so further research needs to be utilized with longitudinal data collection to examine the temporal order of ICC, job stress, and job burnout. Second, the data were based on participants’ self-reports, which may include memory bias or social desirability bias. Third, this study analyzed data that were collected from employees in one case. While the sample size and high response rate increase our confidence, these findings may not be generalizable to the other MNCs. Thus, further studies using random sampling of MNCs are needed.

## 6 Conclusion

This study investigated the associative relations among intercultural communication competence, job stress, and job burnout in a sample of 1,064 employees from a large Chinese MNC based in Brunei. Specifically, we investigated how ICC affects job burnout and whether job stress plays a mediation role between ICC and job burnout. Our findings have demonstrated that ICC has a negative association with job burnout, while job stress is positively related to burnout. In addition, job stress plays a mediating role between ICC and job burnout. This study extends past research by reaching out to employees’ burnout studies under a multinational background and providing evidence of these relations in a sample of Chinese MNC workers. Notably, the results suggest the importance of ICC in work status. Thus, intercultural communication competence interventions are needed to prevent employees from experiencing job burnout.

## Data availability statement

The raw data supporting the conclusions of this article will be made available by the authors, without undue reservation.

## Ethics statement

The studies involving humans were approved by the Review Committee of Research Institute of Social Development, Southwestern University of Finance & Economics. The studies were conducted in accordance with the local legislation and institutional requirements. The participants provided their written informed consent to participate in this study.

## Author contributions

XX: Writing – review & editing, Conceptualization, Data curation, Methodology, Project administration, Resources, Supervision. YT: Investigation, Software, Writing – original draft, Writing – review & editing. CH: Data curation, Formal analysis, Software, Supervision, Validation, Writing – review & editing.

## Footnotes

^1^Ministry of Commerce of the People’s Republic of China, released by Go Global Public Service Platform, release date: 2023-02-13, access date: 2023-04-23, link: http://fec.mofcom.gov.cn/article/fwydyl/tjsj/202302/20230203384457.shtml.

^2^Ministry of Commerce of the People’s Republic of China, released by News Office of the Ministry of Commerce, release date: 2022-11-07, access date: 2023-04-23, link: http://www.mofcom.gov.cn/article/syxwfb/202211/20221103365310.shtml.

^3^Ministry of Commerce of the People’s Republic of China, released by Unified Platform of Business System of Ministry of Commerce, access date: 2023-04-23, link: http://femhzs.mofcom.gov.cn/fecpmvc_zj/pages/fem/CorpJWList.html.

^4^Ministry of Commerce of the People’s Republic of China, released by Foreign Investment and Economic Cooperation Dept., release date: 2023-02-13, access date: 2023-04-23, link: http://www.mofcom.gov.cn/article/tongjiziliao/dgzz/202302/20230203384452.shtml.
