# Corrigendum: Intercultural communication competence and job burnout in MNC employees: the mediation role of job stress

**DOI:** 10.3389/fpsyg.2024.1444048

**Published:** 2024-07-15

**Authors:** Xiaoxia Xie, Yulu Tu, Chienchung Huang

**Affiliations:** ^1^Research Institute of Social Development, Southwestern University of Finance and Economics, Chengdu, China; ^2^Faculty of International Studies, Southwestern University of Finance and Economics, Chengdu, China; ^3^School of Social Work, Rutgers University, New Brunswick, NJ, United States

**Keywords:** Chinese multinational corporation, intercultural communication competence, job burnout, job stress, Southeast Asia

In the published article, there were mistakes in the affiliations of the first author Xiaoxia Xie and the third author Chienchung Huang. In affiliation 1, instead of “Research Institute of Social Development, Southwest University of Finance and Economics, Chengdu, Sichuan Province, China,” it should be “Research Institute of Social Development, Southwestern University of Finance and Economics, Chengdu, China.” In affiliation 3, instead of “School of Social Work, Rutgers University, Newark, NJ, United States,” the correct affiliation should be “School of Social Work, Rutgers University, New Brunswick, NJ, United States.”

The authors apologize for these errors and state that they do not change the scientific conclusions of the article in any way. The original article has been updated.

